# Peptide-Engineered Seliciclib Nanomedicine for Brain-Targeted Delivery and Neuroprotection

**DOI:** 10.3390/ijms26125768

**Published:** 2025-06-16

**Authors:** Guan Zhen He, Wen Jen Lin

**Affiliations:** School of Pharmacy, College of Medicine, National Taiwan University, No. 33, Linsen S. Rd., Taipei 10050, Taiwan; r07423010@ntu.edu.tw

**Keywords:** seliciclib, peptide, nanoparticles, neuroprotection

## Abstract

Seliciclib, a cyclin-dependent kinase 5 (CDK5) inhibitor, has demonstrated neuroprotective potential. However, its therapeutic application is limited by poor permeability across the blood–brain barrier (BBB). In this study, polymeric nanoparticles (NPs) modified with a BBB-targeting peptide ligand (His-Ala-Ile-Tyr-Pro-Arg-His) were employed to encapsulate seliciclib. In vitro transport studies showed that the peptide-modified NPs exhibited significantly greater translocation across a bEnd.3 cell monolayer compared to unmodified NPs. Furthermore, in vivo biodistribution analysis revealed that the brain accumulation of peptide-modified NPs was 3.38-fold higher than that of unmodified NPs. Notably, the peptide-conjugated, seliciclib-loaded NPs demonstrated a significant neuroprotective effect against the neurotoxin 1-methyl-4-phenylpyridinium (MPP⁺) in differentiated SH-SY5Y cells.

## 1. Introduction

Cyclin-dependent kinase 5 (CDK5) involves regulating physiological functions of neurons [1,2]. Under pathological conditions, p25 aberrantly activates CDK5, which results in CDK5 hyperactivation. These aberrant phosphorylation events eventually lead to neurotoxicity and neuronal death [3]. The applications of CDK5 inhibitors in CDK5 related neurodegenerative diseases have been drawing a lot of attention [4,5,6,7]. The blood–brain barrier (BBB) is primarily composed of tightly connected endothelial cells and discontinuous layers of pericytes, which not only control brain homeostasis but also limit the delivery of therapeutic agents to the brain [8]. To overcome this barrier, targeting receptors on the BBB for drug delivery has become a strategy [9,10,11,12]. The transferrin receptor (TfR), a membrane glycoprotein, is highly expressed on the BBB to supply the brain with iron, which is crucial for metabolism and neural conductivity in brain function [13,14,15].

Nanoparticles (NPs) offer several advantages for drug delivery, including protection of therapeutic cargos from degradation, improved pharmacokinetic properties, and reduced recognition by the immune system [16,17,18]. For instance, lipid-based NPs were used in the development of mRNA vaccines such as mRNA-1273 and BNT162b2 for prophylaxis against SARS-CoV-2 infection [19,20]. Similarly, lipid-based NPs encapsulating therapeutic agents have been shown to enhance efficacy in the treatment of Alzheimer’s disease (AD) [21]. Recent research highlights the potential of nanoparticle platforms in the management of central nervous system (CNS) disorders, offering functionalities in drug delivery, neuroprotection, and neuroregeneration. NPs are capable of crossing the blood–brain barrier (BBB), delivering therapeutic agents directly to targeted CNS regions, and improving therapeutic efficacy in models of Alzheimer’s disease, Parkinson’s disease, stroke, and glioblastoma [22]. For example, minocycline (MIN)-loaded Fe_3_O_4_ nanoparticles (Fe_3_O_4_-MIN NPs) have been developed for targeted treatment of Parkinson’s disease. These NPs exhibit BBB-penetrating capabilities and enhance drug delivery to the brain parenchyma, thereby alleviating neuroinflammation and neuronal apoptosis in PD mouse models [23]. Furthermore, surface modification of NPs enables active targeting of overexpressed receptors on the BBB, facilitating efficient drug delivery to brain-specific sites. Cell-penetrating peptides (CPPs) have been employed in the treatment of inflammation, cancer, and CNS disorders due to their capacity to enhance cargo transport across biological barriers, including the BBB [24]. The T7 peptide (HAIYPRH) has been used as an epidermal growth factor receptor active targeting ligand to modify nanocarriers in cancer therapy. Meanwhile, it can bind to the transferrin receptor (TfR) with a dissociation constant of 10 nM. In previous studies, the T7 peptide was shown to facilitate the accumulation of nanoparticles loaded with doxorubicin, siRNA, or their combination at the tumor site in SKOV3 tumor-bearing mice [25,26]. More recently, it has also been applied to deliver the CDK4/6 inhibitor palbociclib to the brain. This delivery system successfully evaded efflux transporters at the BBB and enhanced glioma-targeting capability [27,28]. Notably, T7 peptide does not compete with endogenous transferrin (Tf) or disrupt its physiological functions, as it binds to a different site on TfR [29,30,31]. Moreover, in vivo studies have confirmed that T7 peptide facilitates TfR-mediated uptake, making it a promising targeting ligand for drug delivery in CNS diseases [32].

Seliciclib is a broad-spectrum cyclin-dependent kinase (CDK) inhibitor that has demonstrated therapeutic potential in cancer treatment and neuroprotection, including mitigation of neuronal injury and cognitive dysfunction induced by sevoflurane anesthesia [33]. In our previous study, we developed TfR-targeted seliciclib nanomedicine for cancer therapy. The results showed that T7 peptide-modified nanoparticles achieved improved cellular uptake, particularly in cancer cells with high TfR expression, in the following order: MDA-MB-231 breast cancer cells > SKOV-3 ovarian cancer cells > U87-MG glioma cells [34]. In a rat model of Parkinson’s disease (PD), intrastriatal infusion of seliciclib significantly reduced the severity and amplitude of abnormal involuntary movements (AIMs) associated with L-dopa administration [35]. Moreover, recent studies suggest that seliciclib may ameliorate tauopathies and neuroinflammation, the main pathological features involved in the progression of Alzheimer’s disease (AD) [36,37,38]. However, the clinical utility of seliciclib for brain-related disorders is limited by its high affinity for ATP-binding cassette sub-family B member 1 (ABCB1), a key efflux transporter at the blood–brain barrier (BBB), which restricts its penetration into the central nervous system [39]. Targeting receptors on the BBB, particularly the transferrin receptor (TfR), which is highly expressed on brain capillary endothelial cells (BCECs), has emerged as a potential strategy for delivering drugs into the brain [40].

Therefore, we aimed to develop a peptide-functionalized nanocarrier for the targeted delivery of seliciclib to achieve neuroprotection. This peptide-engineered nanoparticle delivery system was designed to overcome the limitations imposed by the blood–brain barrier (BBB) and facilitate the active transport of seliciclib into the brain. The neuroprotective efficacy of the seliciclib-loaded peptide-conjugated nanoparticles was evaluated using a 1-methyl-4-phenylpyridinium (MPP⁺)-induced parkinsonian SH-SY5Y neuroblastoma cell model. SH-SY5Y cells are widely used in neurodegenerative disease research, particularly in models of Parkinson’s disease. In addition, the brain biodistribution of the peptide-modified nanoparticles was assessed in a mouse model to investigate in vivo targeting capability.

## 2. Results

### 2.1. Characterization of PLGA-PEG-Maleimide Copolymer

**Figure 1:**
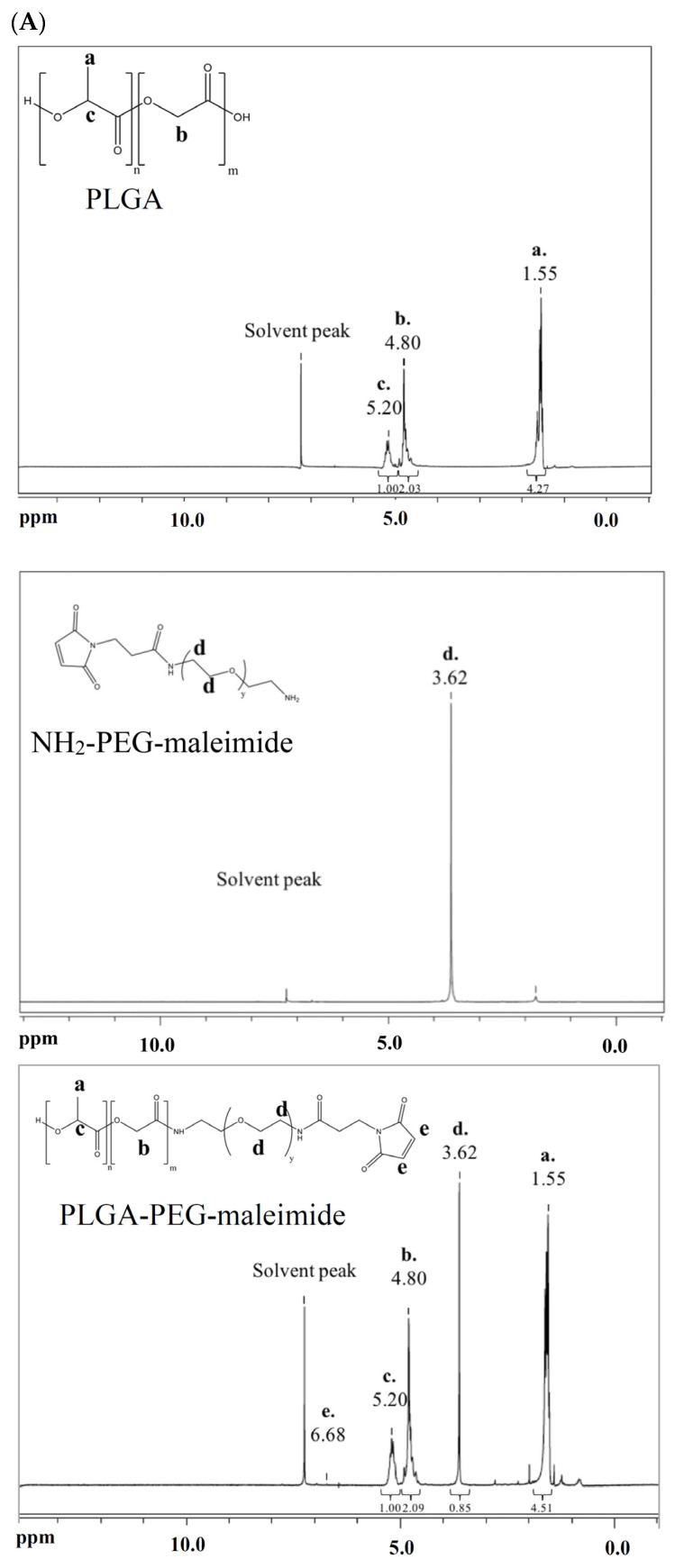

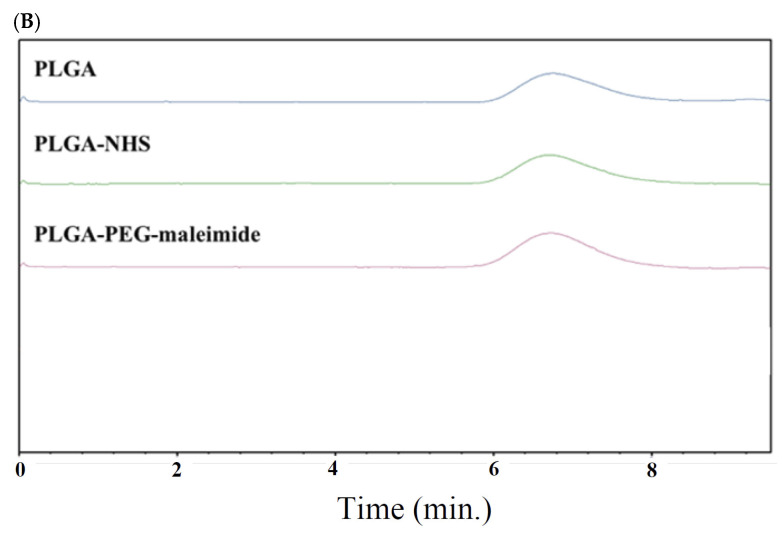
(**A**) Proton nuclear magnetic resonance (^1^H-NMR) spectra and (**B**) size exclusion chromatogram of PLGA-PEG-maleimide.

Figure 1A shows the ^1^H-NMR spectra of PLGA, NH_2_-PEG-maleimide, and the synthesized PLGA-PEG-maleimide. The signals at δ 5.20 ppm, δ 4.80 ppm, and δ 1.55 ppm correspond to PLGA, while the signal at δ 3.62 ppm is attributed to NH_2_-PEG-maleimide. In the spectrum of PLGA-PEG-maleimide, signals derived from both PLGA and NH_2_-PEG-maleimide are simultaneously present, indicating successful conjugation of NH_2_-PEG-maleimide to PLGA. Figure 1B shows the size exclusion chromatograms, where the number-average molecular weight (Mn), weight-average molecular weight (Mw), and polydispersity of PLGA-PEG-maleimide are reported as 32,000 ± 1900 Da, 59,700 ± 2600 Da, and 1.87 ± 0.03, respectively. The pegylation efficiency, calculated using Equation (1), is 60.4 ± 4.0 mol%.

### 2.2. Characterization of Seliciclib@NPs

**Figure 2:**
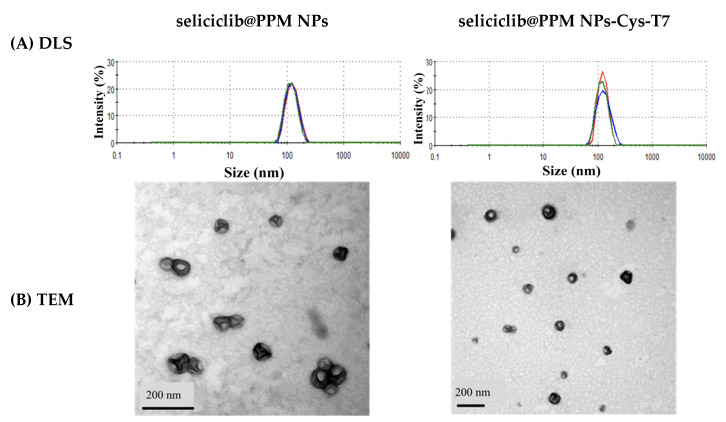
(**A**) Dynamic light scattering (DLS) histograms and (**B**) TEM images of seliciclib@PPM NPs and seliciclib@PPM NPs-Cys-T7.

Seliciclib was encapsulated in PLGA-PEG-maleimide nanoparticles (PPM NPs) to form seliciclib@PPM NPs. These nanoparticles were then modified with the T7 peptide via a maleimide-thiol linkage, resulting in the formation of seliciclib@PPM NPs-Cys-T7. The peptide conjugation efficiency was 26.9 ± 4.8 mol%. Table 1 summarizes the characteristics of both PPM NPs and PPM NPs-Cys-T7 after drug encapsulation. The particle sizes of seliciclib-loaded NPs were in the range of 120–130 nm, with a polydispersity index (PDI) of less than 0.2, indicating a narrow size distribution (Figure 2A). The zeta potential of seliciclib@PPM NPs was changed from −30.8 ± 9.2 mV to −20.0 ± 4.2 mV following T7 peptide conjugation. The encapsulation efficiency and drug loading of seliciclib@PPM NPs and seliciclib@PPM NPs-Cys-T7 were comparable. Transmission electron microscopy (TEM) images revealed that both types of nanoparticles exhibited discrete and spherical morphologies (Figure 2B).

### 2.3. In Vitro Release

**Figure 3:**
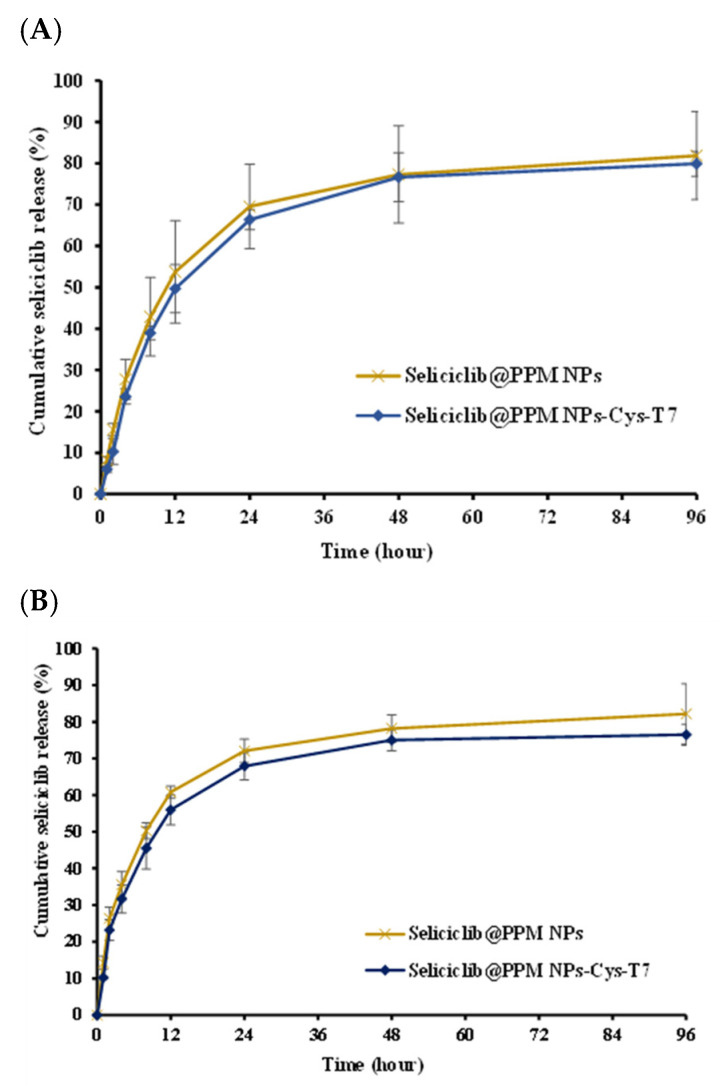
Cumulative release of seliciclib from seliciclib@NPs in (**A**) pH 7.4 phosphate-buffered release medium and (**B**) pH 5.5 acetate-buffered release medium at 37 °C. (*n* = 3, mean ± SD).

Figure 3 shows the release profiles of seliciclib from seliciclib@PPM NPs and seliciclib@PPM NPs-Cys-T7 in phosphate-buffered solution (pH 7.4) and acetate-buffered solution (pH 5.5), simulating physiological conditions and the late endosomal environment, respectively. There were 81.9 ± 10.7% and 79.9 ± 3.0% of seliciclib released from seliciclib@PPM NPs and seliciclib@PPM NPs-Cys-T7, respectively, at 96 h in pH 7.4 medium, with a similarity factor (*f*2) of 70.4. In pH 5.5 medium, 82.2 ± 8.4% and 76.5 ± 2.9% of seliciclib were released from the respective formulations, with an *f*2 value of 67.8.

### 2.4. Transport of Seliciclib@NPs Across BBB Cell Model

**Figure 4:**
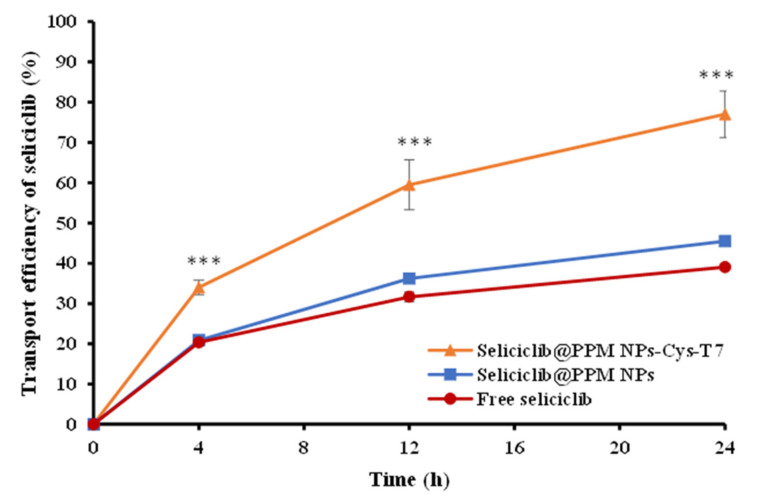
Transport of free seliciclib, seliciclib@PPM NPs, and seliciclib@PPM NPs-Cys-T7 across bEnd.3 monolayer via an in vitro BBB cell model for 24 h. (*n* = 3, mean ± SD, *** *p* < 0.001).

Figure 4 illustrates the transport of seliciclib@PPM NPs and seliciclib@PPM NPs-Cys-T7 across a bEnd.3 cell monolayer in a transwell system, which simulates the physiological blood–brain barrier (BBB) and exhibited a transendothelial electrical resistance (TEER) value of 361.6 ± 13.8 Ω·cm^2^. A slight increase in the transport of seliciclib@PPM NPs was observed compared to free seliciclib; however, the difference was not statistically significant. After 24 h, 45.5 ± 0.5% and 77.0 ± 0.8% of seliciclib were transported across the bEnd.3 monolayer by seliciclib@PPM NPs and seliciclib@PPM NPs-Cys-T7, respectively. Statistical analysis confirmed that the peptide-conjugated nanoparticles achieved significantly higher transport efficiency than the peptide-free ones (*p* < 0.001).

### 2.5. Cellular Uptake of Nanoparticles

**Figure 5:**
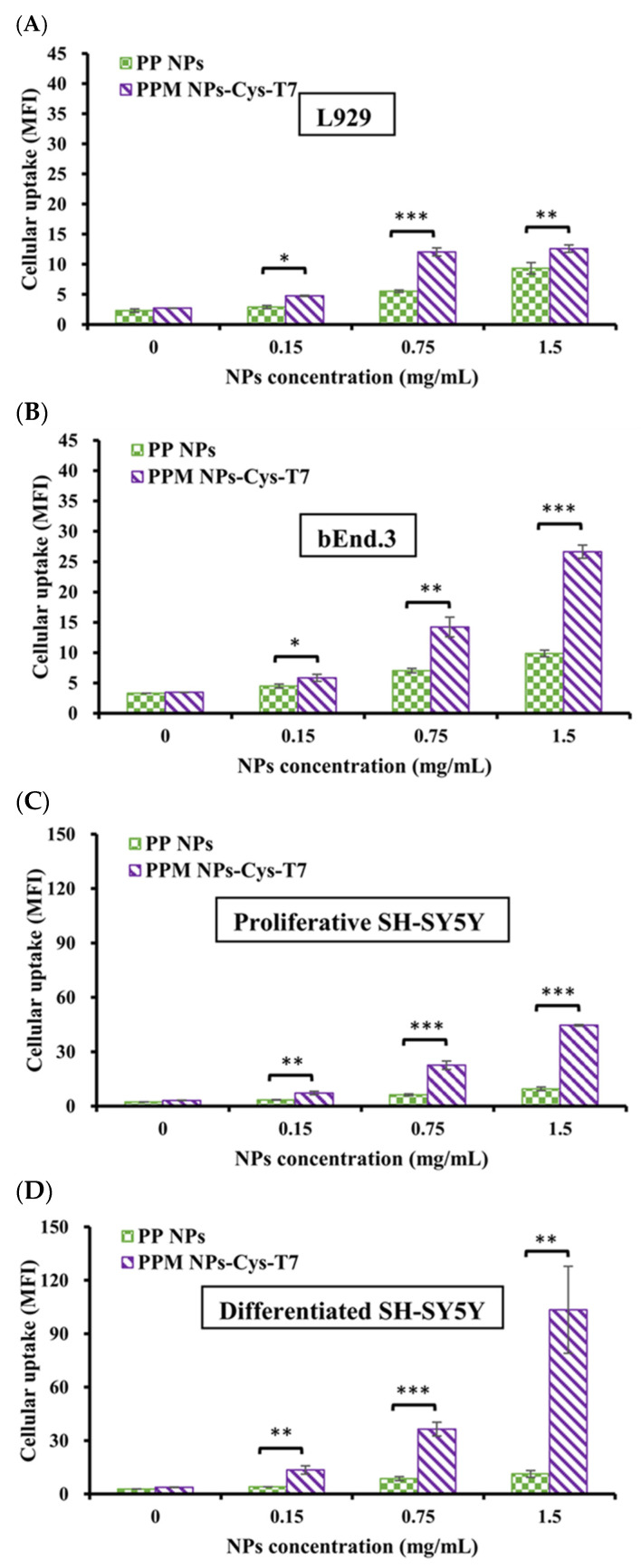

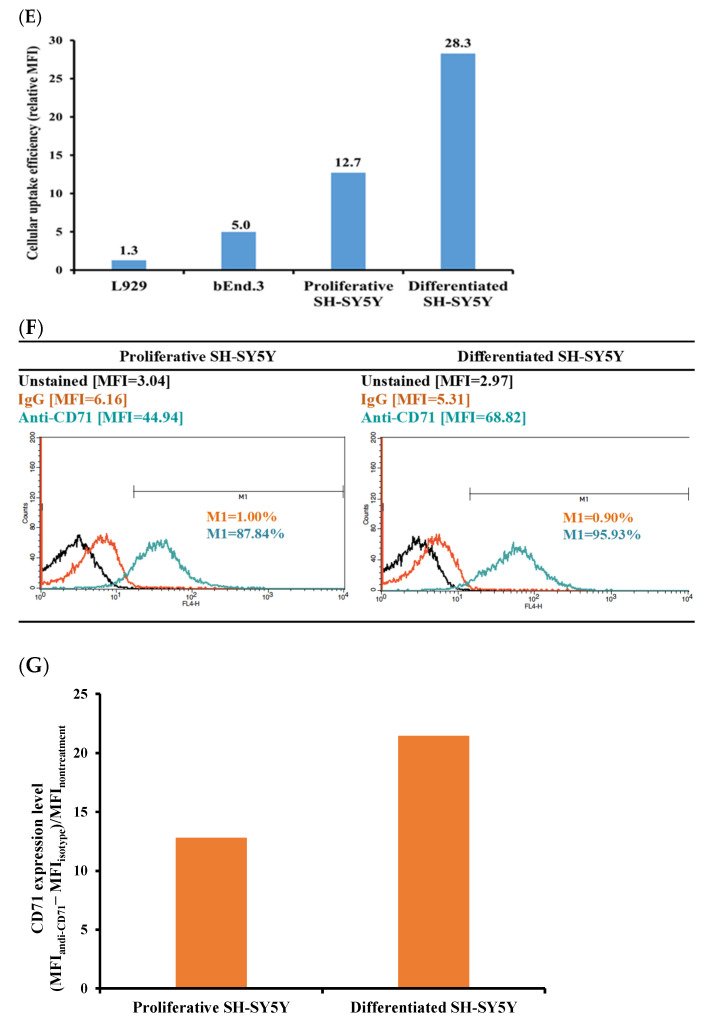
Cellular uptake of peptide-free PP NPs and peptide-conjugated PPM NPs-Cys-T7 in (**A**) L929 fibroblast cells (as control), (**B**) bEnd.3 endothelial cells, (**C**) proliferative SH-SY5Y cells, as well as (**D**) differentiated SH-SY5Y cells under 5% CO_2_ at 37 °C for 2 h analyzed by flow cytometry (*n* = 3, mean ± SD, * *p* < 0.05, ** *p* < 0.01, *** *p* < 0.001, compared to PP NPs), and (**E**) the increase in cellular uptake of peptide-conjugated NPs relative to peptide-free NPs (calculated by Equation (4)) at 1.5 mg/mL in four cell lines. (**F**) Flow cytometric analysis of MFI on proliferative and differentiated SH-SY5Y cells stained with anti-human CD71 antibody (green line) and isotype IgG control (orange line), and (**G**) their CD71 expression levels calculated by Equation (3).

Figure 5A–D illustrate the cellular uptake of peptide-free PLGA-PEG nanoparticles (PP NPs) and peptide-conjugated PPM NPs-Cys-T7 in various cell lines, including L929 fibroblasts (negative control), bEnd.3 endothelial cells, and both proliferative and differentiated SH-SY5Y neuroblastoma cells, following a 2 h incubation at 37 °C. L929 cells, which express low levels of the transferrin receptor (CD71), exhibited minimal uptake, even at the highest concentration (1.5 mg/mL) of PPM NPs-Cys-T7 (Figure 5A). In contrast, bEnd.3 cells demonstrated higher uptake of the peptide-conjugated nanoparticles compared to L929 cells, with significantly greater mean fluorescence intensity (MFI) than peptide-free PP NPs (Figure 5B). Uptake was further enhanced in proliferative SH-SY5Y cells (Figure 5C) and peaked in differentiated SH-SY5Y cells (Figure 5D), particularly at 1.5 mg/mL.

Figure 5E summarizes the fold increase in cellular uptake of peptide-conjugated PPM NPs-Cys-T7 relative to peptide-free PP NPs across these cell lines. In L929 cells, the relative uptake ratio showed only a 1.3-fold increase, whereas bEnd.3 cells exhibited a 5-fold enhancement. Notably, uptake was significantly enhanced in SH-SY5Y cells, with a 12.7-fold increase in proliferative cells and a 28.3-fold increase in differentiated cells. This pronounced enhancement in cellular uptake strongly correlated with CD71 expression levels, as shown in Figure 5F–G.

### 2.6. Brain Biodistribution of Nanoparticles by IVIS

**Figure 6:**
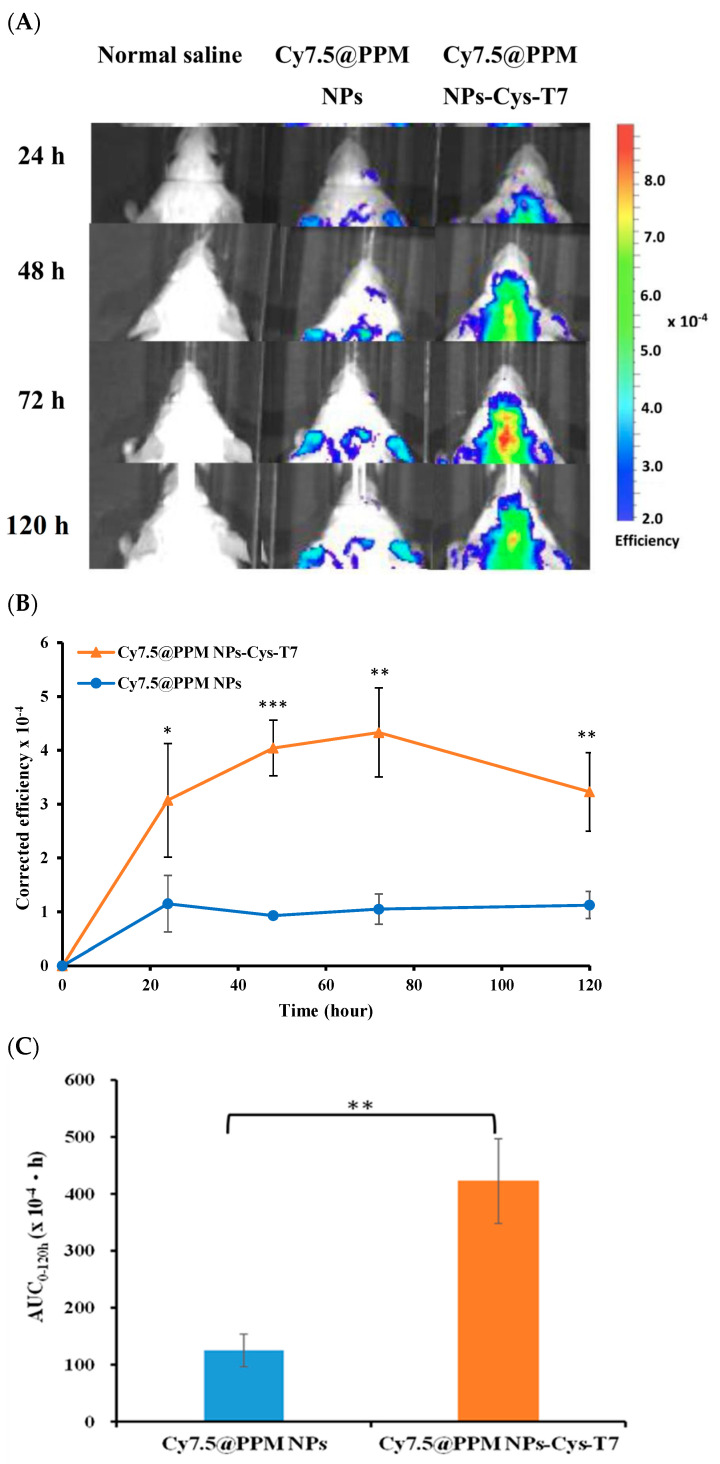

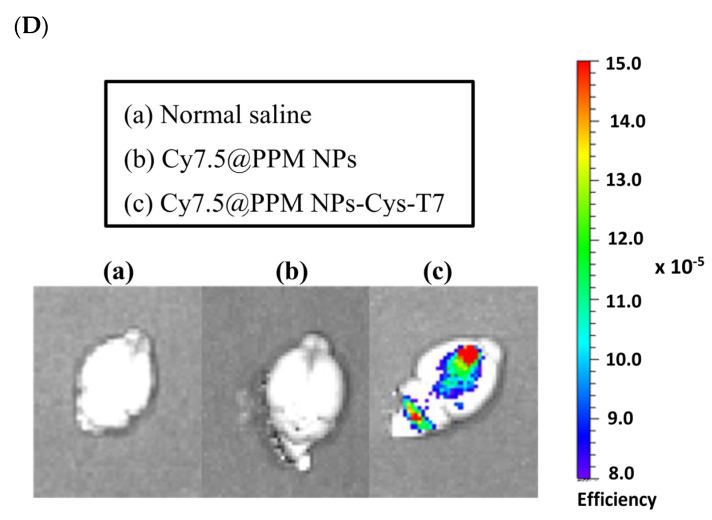
(**A**) IVIS images of Cy7.5@PPM NPs and Cy7.5@PPM NPs-Cys-T7 in mice brain over 5-day period following intravenous injection via the tail vein. (**B**) The fluorescence intensity versus time profiles of Cy7.5@NPs accumulated in the brain, and (**C**) the corresponding area under the curve AUC_0–120 h_. (**D**) Ex vivo IVIS images of brains collected 72 h post-injection (*n* = 3, mean ± SD, * *p* < 0.05, ** *p* < 0.01, *** *p* < 0.001).

Figure 6 illustrates the brain biodistribution of peptide-free Cy7.5@PPM NPs and peptide-conjugated Cy7.5@PPM NPs-Cys-T7 in mice over a 5-day period following intravenous injection via the tail vein. The IVIS images show that Cy7.5@PPM NPs-Cys-T7 accumulated significantly more in the brain region compared to Cy7.5@PPM NPs (Figure 6A). The fluorescence intensity of Cy7.5@NPs in the brain over time is presented in Figure 6B, and the corresponding area under the curve (AUC_0–120 h_) is 125.2 ± 28.8 (×10^4^·h) for Cy7.5@PPM NPs and 422.4 ± 74.4 (×10^4^·h) for Cy7.5@PPM NPs-Cys-T7 (Figure 6C). Ex vivo IVIS images of Cy7.5@NPs in the brain were further obtained after the mice were euthanized at 72 h post-injection (Figure 6D). A markedly stronger signal was observed in the brain following administration of Cy7.5@PPM NPs-Cys-T7, representing a 3.4-fold increase compared to Cy7.5@PPM NPs.

### 2.7. Neuroprotection Effect in MPP^+^-Induced Parkinsonian SH-SY5Y Cell Model

**Figure 7:**
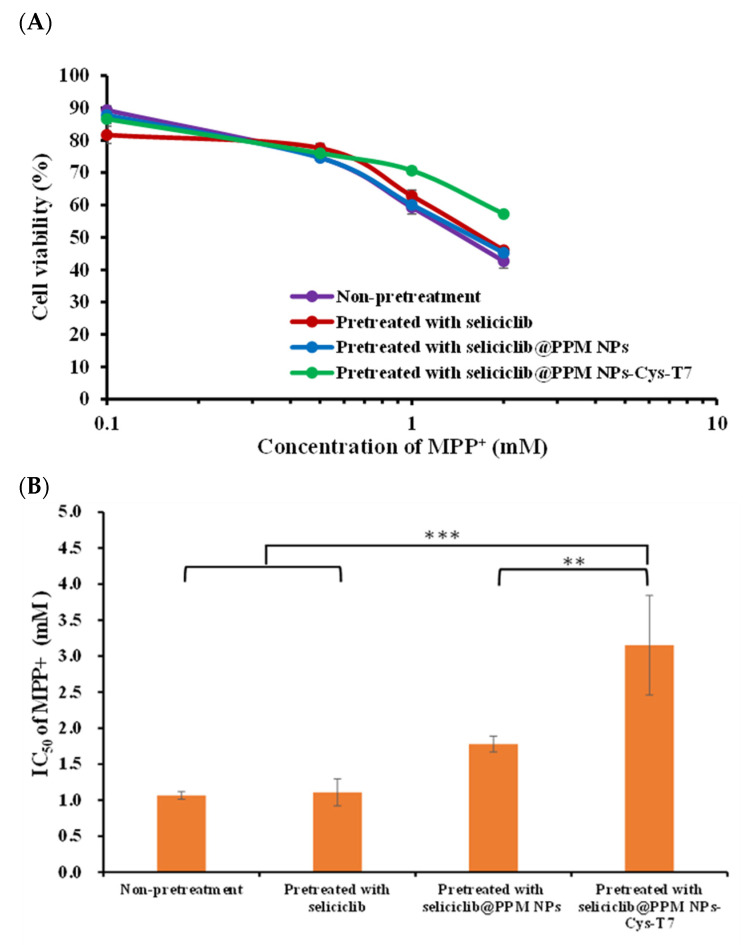
(**A**) Cell viability of differentiated SH-SY5Y pretreated with 10 μg/mL of seliciclib, seliciclib@PPM NPs or seliciclib@PPM NPs-Cys-T7 for 2 h, followed by incubation with various concentrations of MPP^+^ (0.1–2.0 mM) for 48 h, and (**B**) the corresponding IC_50_ values were 1.11 ± 0.19 mM, 1.78 ± 0.11 mM and 3.15 ± 0.69 mM, respectively. (*n* = 3, mean ± SD, ** *p* < 0.01, *** *p* < 0.001).

The positively charged 1-methyl-4-phenylpyridinium (MPP⁺) has been widely used in Parkinson’s disease-related studies and acts as a neurotoxin by interfering with oxidative phosphorylation in mitochondria, ultimately leading to cell death. In this study, the neuroprotective effect of seliciclib-loaded nanoparticles (seliciclib@NPs) against MPP⁺-induced cytotoxicity was evaluated in differentiated SH-SY5Y cells. The cells were pretreated with free seliciclib, seliciclib@PPM NPs, or seliciclib@PPM NPs-Cys-T7 for 2 h, followed by incubation with various concentrations of MPP⁺ (0.1–2.0 mM) at 37 °C for 48 h. Cell viability was subsequently assessed by the MTT method (Figure 7A), and the corresponding IC_50_ values were 1.11 ± 0.19 mM, 1.78 ± 0.11 mM, and 3.15 ± 0.69 mM, respectively (Figure 7B). The most pronounced neuroprotection against MPP⁺-induced cytotoxicity was observed in peptide-conjugated seliciclib@PPM NPs-Cys-T7, which demonstrated a 2.8-fold (*p* < 0.001) and 1.8-fold (*p* < 0.01) increase in protective efficacy compared to the free drug and peptide-free seliciclib@PPM NPs, respectively.

## 3. Discussion

Seliciclib is a broad-spectrum CDK inhibitor that has demonstrated therapeutic potential in cancer treatment and neuroprotection [33]. In a rat model of Parkinson’s disease (PD), intrastriatal infusion of seliciclib significantly reduced the severity and amplitude of abnormal involuntary movements (AIMs) associated with L-dopa administration [35]. Moreover, recent studies suggest that seliciclib may ameliorate tauopathies and neuroinflammation, which are key pathological features involved in the progression of Alzheimer’s disease (AD) [36,37,38]. However, the clinical utility of seliciclib for brain-related disorders is limited by its high affinity for ATP-binding cassette sub-family B member 1 (ABCB1), a major efflux transporter at the blood–brain barrier (BBB), which restricts its penetration into the central nervous system [39]. To overcome this limitation, a CD71-targeted nanocarrier system was developed to facilitate seliciclib delivery. We used an FDA-approved and widely used biodegradable PLGA copolymer with pegylation to prolong the in vivo circulation time [41]. The ^1^H-NMR spectra provide evidence of successful conjugation of PEG to PLGA, with a pegylation efficiency of 60.4 ± 4.0 mol%.

Furthermore, a T7 peptide-modified nanoparticle platform was designed to overcome the limitations imposed by the blood–brain barrier (BBB) and to facilitate the active transport of seliciclib into the brain. T7 peptide (His-Ala-Ile-Tyr-Pro-Arg-His) is a seven-amino-acid peptide with a high affinity for the human transferrin receptor (TfR) [31]. Studies have shown that the expression levels of TfR in brain endothelial cells and glioma cancer cells are higher than in normal cells, making T7 peptide a promising candidate for targeted drug delivery [28,42,43]. Seliciclib-encapsulated nanoparticles (seliciclib@PPM NPs) were modified with the T7 peptide via a maleimide-thiol linkage. The peptide conjugation efficiency of seliciclib@PPM NPs-Cys-T7 was 26.9 ± 4.8 mol%. The seliciclib@NPs, with or without peptide modification, exhibited particle sizes in the range of 120–130 nm with a narrow size distribution. This was supported by TEM images, which revealed that both types of nanoparticles exhibited discrete and spherical morphologies. The zeta potential of seliciclib@PPM NPs shifted from −30.8 ± 9.2 mV to −20.0 ± 4.2 mV following T7 peptide conjugation, likely due to the presence of positively charged arginine and histidine residues in the peptide. Both seliciclib@PPM NPs and seliciclib@PPM NPs-Cys-T7 exhibited comparable drug loading capacities.

The release of seliciclib from seliciclib@PPM NPs and seliciclib@PPM NPs-Cys-T7 was evaluated in buffered solutions at pH 7.4 and pH 5.5, simulating physiological conditions and the late endosomal environment, respectively. Seliciclib@NPs exhibited a prolonged-release profile, and the conjugation of the peptide ligand did not significantly alter the release behavior of seliciclib from the nanoparticles, as indicated by *f*_2_ values greater than 50.

The transport of peptide-free seliciclib@PPM NPs and peptide-conjugated seliciclib@PPM NPs-Cys-T7 was evaluated using a transwell system with bEnd.3 cells to simulate the physiological blood–brain barrier (BBB). No statistically significant difference was observed between the transport of seliciclib@PPM NPs and free seliciclib, although the former exhibited slightly higher transport. In contrast, the peptide-conjugated seliciclib@PPM NPs-Cys-T7 demonstrated significantly greater transport efficiency (77.0 ± 0.8%) compared to peptide-free seliciclib@PPM NPs (45.5 ± 0.5%) at 24 h (*p* < 0.001). These results confirm that the T7 peptide facilitates seliciclib delivery across the bEnd.3 monolayer, surpassing the inherent enhanced permeability and retention (EPR) effect associated with nano-sized nanoparticles.

The cellular uptake capability of peptide-free PLGA-PEG nanoparticles (PP NPs) and peptide-conjugated PPM NPs-Cys-T7 was illustrated in several cell lines, including bEnd.3 endothelial cells to simulate the physiological BBB, SH-SY5Y neuroblastoma cells commonly used in studies of neurodegenerative diseases, and L929 fibroblast cells as a negative control. Cells were incubated with the nanoparticles at 37 °C for 2 h. The relative cellular uptake efficiency of peptide-conjugated PPM NPs-Cys-T7 compared to peptide-free PP NPs followed the order: L929 (1.3×) < bEnd.3 (5.0×) < proliferative SH-SY5Y (12.7×) < differentiated SH-SY5Y (28.3×). L929 cells, which express low levels of CD71, exhibited the lowest cellular uptake among these cell lines. In bEnd.3 cells, peptide conjugation increased nanoparticle uptake fivefold, suggesting involvement of transferrin receptor (CD71)-mediated endocytosis. Notably, the cellular uptake of PPM NPs-Cys-T7 was enhanced by 12.7-fold and 28.3-fold in proliferative and differentiated SH-SY5Y cells, respectively. Differentiated SH-SY5Y cells with a mature, neuron-like phenotype were obtained by culturing the proliferative cells in DMEM/F12 with 1% fetal bovine serum and treating them with 10 μM retinoic acid for 7 days [44]. The observed differences in cellular uptake between proliferative and differentiated SH-SY5Y cells strongly correlated with CD71 expression levels, highlighting the critical role of the peptide ligand in enhancing the targeted uptake of PPM NPs-Cys-T7, particularly in mature neurons with higher CD71 expression.

The in vivo brain distribution of peptide-free Cy7.5@PPM NPs and peptide-conjugated Cy7.5@PPM NPs-Cys-T7 was investigated in a mouse model following intravenous injection via the tail vein. Quantitative analysis of IVIS fluorescence imaging over a 5-day period revealed that Cy7.5@PPM NPs-Cys-T7 exhibited significantly greater accumulation in the brain compared to peptide-free Cy7.5@PPM NPs (*p* < 0.01). Ex vivo IVIS imaging of brain tissues at 72 h post-injection further confirmed markedly stronger fluorescence signals in mice treated with the peptide-conjugated nanoparticles. The striatum has been reported to be a region enriched with tyrosine hydroxylase (TH)-expressing dopaminergic neurons, which are implicated in the pathogenesis of Parkinson’s disease (PD) [45,46]. Additional imaging of brain sections using fluorescence microscopy or immunohistochemistry could provide further evidence for identifying key target regions within the brain. Collectively, these findings confirm that the T7 peptide effectively facilitates the penetration of Cy7.5@PPM NPs-Cys-T7 across the blood–brain barrier (BBB) and enables targeted delivery to PD-relevant brain regions [47].

The positively charged 1-methyl-4-phenylpyridinium (MPP⁺) has been widely used in Parkinson’s disease-related studies and functions as a neurotoxin by disrupting oxidative phosphorylation in mitochondria, ultimately leading to cell death [48]. In this study, the neuroprotective effect of seliciclib-loaded nanoparticles (seliciclib@NPs) against MPP⁺-induced cytotoxicity was elucidated in differentiated SH-SY5Y cells. The cells were pretreated with free seliciclib, seliciclib@PPM NPs, or seliciclib@PPM NPs-Cys-T7 for 2 h, followed by incubation with various concentrations of MPP⁺ (0.1–2.0 mM) at 37 °C for 48 h. The protective effect of seliciclib@NPs against MPP⁺-induced cytotoxicity was assessed by measuring cell viability using the MTT assay. The corresponding IC_50_ values were as follows: free seliciclib (1.11 ± 0.19 mM) < seliciclib@PPM NPs (1.78 ± 0.11 mM) < seliciclib@PPM NPs-Cys-T7 (3.15 ± 0.69 mM). Seliciclib@PPM NPs-Cys-T7 exhibited significantly greater protective efficacy compared to free seliciclib (*p* < 0.001) and peptide-free seliciclib@PPM NPs (*p* < 0.01). These results indicate that the seliciclib-loaded peptide-conjugated NPs provide the most pronounced neuroprotection against MPP⁺-induced cytotoxicity. It would be worthwhile to evaluate the neuroprotective effects of our nanoparticles in 6-OHDA- or MPTP-induced Parkinson’s disease animal models to validate their in vivo efficacy in future studies [49].

In addition to the enhanced brain accumulation demonstrated by the peptide-modified polymeric nanomedicine in our study, which highlights its potential as a nanocarrier for targeted brain delivery, various other strategies have also been explored to improve blood–brain barrier (BBB) targeting and neuroprotective efficacy. For instance, lipid nanoparticles have been shown to enhance BBB penetration and improve the neuroprotective effect of Ferrostatin-1 against cerebral ischemic injury [50]. Azouz et al. reported that the neuroprotective efficacy of cinnamaldehyde was enhanced by cationic lecithin-based nanocomposites, which downregulated Aβ_1-42_ and phosphorylated tau (p-tau) in cerebral tissues [51]. Furthermore, Xu et al. developed a thiolated gelatin/hyaluronan hydrogel co-loaded with chondroitinase ABC (ChABC) and IGF-1, which provided both neuroprotection and neuroregeneration in the treatment of intracerebral hemorrhage [52].

## 4. Materials and Methods

### 4.1. Materials

Poly(D,L-lactide-co-glycolide) 50:50 (PLGA, 52,000 g/mol) was ordered from Evonik Industries (Birmingham, AL, USA). Maleimide poly(ethylene glycol) amine (Mal-PEG-amine, 5000 g/mol) was obtained from Hunan Hua Teng Pharmaceutical Co., Ltd. (Merelbeke, Belgium). Thiazolyl blue tetrazolium bromide (MTT, 98%) was purchased from Alfa Aesar (Echo Chemical Co., Ltd., Heysham, UK). FITC-Cys-T7 peptide was obtained from Kelowna International Scientific Inc. (Taipei, Taiwan). FITC-NHS was obtained from Thermo Fisher Scientific Inc. (Hudson, NH, USA). Rat IgG2a kappa isotype control APC, mouse IgG1 kappa isotype control APC, and anti-mouse as well as anti-human CD71 monoclonal antibody allophycocyanin (APC) were obtained from eBioscience, Inc. (Vienna, Austria). Retinoic acid (ATRA) was obtained from AdooQ Bioscience (Irvine, CA, USA). 1-Methyl-4-phenylpyridinium (MPP^+^) iodide was obtained from Cayman Chemical (Ann Arbor, MI, USA). Cyanine7.5 NHS ester was purchased from Lumiprobe Corporation (Hallandale Beach, FL, USA). Seliciclib was obtained from LC Laboratories (Woburn, MA, USA). L929 and bEnd.3 cell lines were ordered from Bioresource Collection and Research Center (Hsinchu, Taiwan). The SH-SY5Y cell line (ATCC^®^CRL-2266) was obtained from ATCC (Manassas, VA, USA). Zetasizer was from Malvern Instruments (Nano-ZS90, Worcestershire, UK). HPLC and a UV detector were from Jasco International Co., Ltd. (Tokyo, Japan). Transmission electron microscopy (TEM) was from Hitachi High-Technologies Corporation (Hitachi H7650, Tokyo, Japan). FACSCalibur flow cytometer was from Becton Dickinson (Franklin Lakes, NJ, USA) with BD CellQuest^TM^ Pro software (version 6.0, San Jose, CA, USA). In vivo imaging system (IVIS) was from Xenogen Corporation (Imaging system 200 series, Alameda, CA, USA). SigmaPlot 12.5 software was from Softhome International, Inc. (Taipei, Taiwan).

### 4.2. Synthesis and Characterization of Copolymers

PLGA-PEG-maleimide copolymer was synthesized. PLGA was pre-activated to PLGA-NHS in the presence of NHS and EDC, followed by pegylation with NH_2_-PEG-maleimide. PLGA-NHS and NH_2_-PEG-maleimide (molar ratio 1:2) were dissolved in chloroform and reacted for 24 h in the dark [53,54]. The resulting product was precipitated using a mixture of ice-cold methanol and diethyl ether (1:4 *v*/*v*) and then centrifuged. After discarding the supernatant, the product was re-dissolved in chloroform. Finally, PLGA-PEG-maleimide was dried and stored at −20 °C. The molecular weight was determined by size exclusion chromatography (SEC) equipped with a refractive index detector. A Styragel^®^ HR 4E column (Waters, Milford, MA, USA) was used, with HPLC-grade chloroform as the mobile phase. Prior to injection, the polymer sample was filtered through a 0.22 µm polytetrafluoroethylene (PTFE) membrane. The molecular weight and polydispersity of the copolymer were calculated based on a calibration curve established using polystyrene standards. The pegylation efficiency was determined by integrating the signals from proton nuclear magnetic resonance (^1^H-NMR) spectra according to Equation (1).

(1) $$\mathrm{pegylation}\;\mathrm{efficiency}\;(\mathrm{mol}\%)=\frac{\frac{Area\;(3.62\;ppm)}{4\;\times \frac{MW\;of\;PEG}{MW\;of\;EG\;monomer}}}{\frac{Area\;\left(1.55\;ppm\right)+Area\;\left(4.80\;ppm\right)+Area\;(5.20\;ppm)}{6\;\times \;\frac{MW\;of\;PLGA}{MW\;of\;(LA\;monomer\;+GA\;monomer)}}}\times 100\%$$

### 4.3. Preparation and Characterization of Nanoparticles

NPs encapsulating seliciclib (seliciclib@NPs) were prepared using the solvent evaporation method [55]. Seliciclib and PLGA-PEG-maleimide copolymer were weighed at a weight ratio of 1:5 and dissolved in dichloromethane and then added to a phosphate-buffered saline solution (PBS, pH 7.4) containing 0.5% PVA (o/w 1:10 *v*/*v*). The resulting mixture was sonicated in an ice bath and stirred magnetically for 4 h. After removal of the residual organic solvent, seliciclib@PPM NPs were collected after centrifugation. Furthermore, drug-loaded peptide-conjugated NPs (seliciclib@PPM NPs-Cys-T7) were prepared. The seliciclib@PPM NPs suspension was reacted with the Cys-T7 peptide (molar ratio of 1:2) in PBS (pH 7.4) at room temperature for 2 h. The seliciclib@PPM NPs-Cys-T7 were then collected after centrifugation and stored at 4°C. The zeta potential (ZP), particle size, and polydispersity index (PDI) of seliciclib@NPs were measured using a zetasizer. The payload of seliciclib was determined by HPLC using an SCpak ODS-P C18 column (4.6 × 250 mm, 5 μm, Bouc-Bel-Air, France) and a UV detector set at 290 nm. The mobile phase consisted of acetonitrile and 0.01 M sodium dihydrogen phosphate buffer at a ratio of 7:3 (*v*/*v*), with a flow rate of 1.0 mL/min. The encapsulation efficiency (EE) and drug loading (DL) were calculated. The fluorescence of fluorescein isothiocyanate (FITC)-labeled T7-peptide was measured (λ_ex_ = 485 nm, λ_em_ = 535 nm), and the conjugation ratio of T7-peptide was calculated using Equation (2). In addition, the morphology of the NPs was observed by TEM.

(2) $$Peptide\;conjugation\;efficiency\;(mol\%)\;=\;\frac{\frac{Conc.\;of\;FITC\;labeled\;Cys\;-\;T7\;peptide}{MW\;of\;FITC\;labeled\;Cys\;-\;T7\;peptide\;(1498.71\;g/mol)}}{\frac{Conc.\;of\;PLGA\;-\;PEG\;-\;maleimide}{MW\;of\;PLGA\;-\;PEG\;-\;maleimide\;(59700\;g/mol)}}$$

### 4.4. Transport of Seliciclib@NPs Across BBB Cell Model

The bEnd.3 endothelial cells were uniformly seeded in the upper insert of a Transwell system (0.4 µm pore size, surface area 4.52 cm^2^, PET membrane, SPL) at a density of 2 × 10^5^ cells/well, and cultured for four days to establish an intact bEnd.3 monolayer with a transendothelial electrical resistance (TEER) value of 361.6 ± 13.8 Ω·cm^2^ [56,57,58]. The transport of seliciclib@NPs across the bEnd.3 cell barrier was then investigated. Seliciclib@PPM NPs and seliciclib@PPM NPs-Cys-T7 were dispersed in serum-free DMEM and added to the upper insert of the Transwell system, while fresh DMEM was added to the lower chamber. At predetermined time points, samples were collected from the lower chamber and replaced with an equal volume of fresh DMEM. The collected samples were centrifuged, and the drug concentration was quantified by high-performance liquid chromatography (HPLC).

### 4.5. Identification of CD71 Expression Level

The expression of CD71 on L929, bEnd.3, and SH-SY5Y cells was evaluated using an isotype control APC and an allophycocyanin (APC)-conjugated anti-CD71 monoclonal antibody. Cells were suspended in staining buffer at a concentration of 1 × 10^6^ cells/mL. Either the isotype control APC or the anti-CD71 antibody was added to the cell suspension and incubated for one hour at room temperature. Following incubation, the cells were centrifuged, washed three times with staining buffer, and resuspended in the same buffer for analysis using a FACSCalibur flow cytometer. The upper limit of the isotype control group, representing nonspecific binding, was set to <1% of total events. A total of 10,000 events were acquired. The percentage of M1-gated cells and the mean fluorescence intensity (MFI) were recorded, and the relative mean fluorescence intensity (MFI), representing CD71 expression, was calculated using Equation (3).

(3) $$Relative\;MFI=\frac{\left({MFI}_{anti-CD71}-{MFI}_{isotype}\right)}{{MFI}_{nontreatment}}$$

### 4.6. In Vitro Cellular Uptake of Nanoparticles

The cellular uptake of peptide-free PLGA-PEG nanoparticles (PP NPs) and peptide-conjugated PPM NPs-Cys-T7 was evaluated in L929 and bEnd.3 cells, as well as in proliferative and differentiated SH-SY5Y cells, by measuring the mean fluorescence intensity (MFI) of the fluorescent probe FITC. L929 and bEnd.3 cells were seeded in 24-well plates at a density of 2 × 10^5^ cells/well in DMEM supplemented with 10% fetal bovine serum and 1% penicillin-streptomycin-amphotericin B (PSA) and incubated for 24 h. Proliferative (undifferentiated) SH-SY5Y cells were cultured under the same conditions, except that DMEM/F12 was used as the medium. To obtain differentiated SH-SY5Y cells with a mature, neuron-like phenotype, proliferative SH-SY5Y cells were cultured in DMEM/F12 containing 1% fetal bovine serum and treated with 10 µM retinoic acid for 7 days. PPM NPs-Cys-T7 and PP NPs were then incubated with L929, bEnd.3, and both proliferative and differentiated SH-SY5Y cells at 37 °C for 2 h. After incubation, the cells were washed three times with PBS, trypsinized, centrifuged, and collected for analysis using a FACSCalibur flow cytometer. The increase in cellular uptake of peptide-modified PPM NPs-Cys-T7 relative to peptide-free PP NPs was calculated using Equation (4).

(4) $$Relative\;cellular\;uptake=\frac{{MFI}_{PPM\;NPs-Cys-T7}-{MFI}_{PP\;NPs}}{{MFI}_{nontreatment}}$$

### 4.7. Brain Biodistribution of Nanoparticles by IVIS

All animal experiments were approved by the Institutional Animal Care and Use Committee of National Taiwan University and were conducted in accordance with the National Research Council’s Guide for the Care and Use of Laboratory Animals. Male BALB/c mice (7 weeks old, 20–22 g; BioLASCO, Yilan, Taiwan) were used to evaluate the in vivo biodistribution of fluorescently labeled Cy7.5@PPM NPs and Cy7.5@PPM NPs-Cys-T7 [59]. The mice were intravenously injected via the tail vein with Cy7.5@NPs, while the control group received an injection of normal saline. In vivo fluorescence imaging was performed at predetermined time points using an IVIS imaging system with excitation at 710 nm and emission at 820 nm. After 72 h, the mice were euthanized, and ex vivo images of the brains were acquired. The corresponding fluorescence intensities were normalized to the surface area of the brain in each image, and the specific uptake efficiency was calculated using Equation (5).

(5) $$Specific\;efficiency=\frac{\left({Efficiency}_{Cy7.5@NPs}-{Efficiency}_{normal\;saline}\right)}{Surface\;area\;of\;brain\;in\;the\;image}$$

### 4.8. Neuroprotective Effect in MPP^+^-Induced Parkinsonian SH-SY5Y Cell Model

Differentiated SH-SY5Y cells were seeded uniformly in a 96-well plate at a density of 2 × 10^4^ cells/well in DMEM/F12 medium supplemented with 10% bovine growth serum and 1% penicillin-streptomycin-amphotericin B (PSA) and incubated for 48 h. The medium was then replaced with 100 µL of either free seliciclib (10 µg/mL) or seliciclib@NPs in DMEM/F12, followed by incubation for 2 h. Subsequently, the medium was removed, and various concentrations of MPP⁺ (0.1–2.0 mM) were added and incubated at 37 °C for 48 h. MTT solution was then added to each well and incubated for an additional 4 h. The resulting formazan crystals were solubilized in dimethyl sulfoxide, and absorbance was measured at 570 nm and 690 nm using a microplate reader (SpectraMax Paradigm, San Jose, CA, USA). Cell viability was calculated using Equation (6).

(6) $$Cellular\;viability(\%)=\frac{{\left[{OD}_{570\;nm}-{OD}_{690\;nm}\right]}_{sample}}{{\left[{OD}_{570\;nm}-{OD}_{690\;nm}\right]}_{control}}\times 100\%$$

### 4.9. Statistical Analysis

All statistics was performed using SigmaPlot 12.5 software (Softhome International, Inc., Taipei, Taiwan). One-way analysis of variance (ANOVA) and unpaired Student’s *t*-test were applied. The statistical significance was defined as *p* < 0.05.

## 5. Conclusions

The neuroprotective potential of seliciclib nanomedicine was demonstrated through both in vitro and in vivo studies. Conjugation with the T7 peptide enhanced the targeting capability of seliciclib@PPM NPs-Cys-T7, facilitating improved penetration across a bEnd.3 monolayer in an in vitro BBB model and increasing cellular uptake, particularly in differentiated SH-SY5Y cells. The peptide-modified seliciclib@PPM NPs-Cys-T7 exhibited superior neuroprotection against the neurotoxin MPP⁺ compared to both the free drug and peptide-free seliciclib@PPM NPs. Furthermore, the better accumulation of peptide-conjugated NPs in the brain highlights their potential as a promising nanocarrier for targeted brain delivery.

## Figures and Tables

**Table 1:** Characteristics of seliciclib-loaded PPM NPs and PPM NPs-Cys-T7 (*n* = 3, mean ± SD).

Formulations	Size (nm)	PDI	ZP (mV)	Yield (%)	EE (%)	DL (%)
Seliciclib@PPM NPs	115.7 ± 5.5	0.11 ± 0.03	−30.8 ± 9.2	72.5 ± 3.6	64.8 ± 3.7	14.9 ± 1.0
Seliciclib@PPM NPs-Cys-T7	127.3 ± 0.7	0.19 ± 0.03	−20.0 ± 4.2	81.3 ± 1.7	60.0 ± 1.2	12.3 ± 0.5

## Data Availability

Data were generated during the study.
